# MDAT- Aligning multiple domain arrangements

**DOI:** 10.1186/s12859-014-0442-7

**Published:** 2015-01-28

**Authors:** Carsten Kemena, Tristan Bitard-Feildel, Erich Bornberg-Bauer

**Affiliations:** Institute for Evolution and Biodiversity, University of Münster, Hüfferstr. 1, Münster, Germany

**Keywords:** Domain arrangement, Multiple alignment

## Abstract

**Background:**

Proteins are composed of domains, protein segments that fold independently from the rest of the protein and have a specific function. During evolution the arrangement of domains can change: domains are gained, lost or their order is rearranged. To facilitate the analysis of these changes we propose the use of multiple domain alignments.

**Results:**

We developed an alignment program, called MDAT, which aligns multiple domain arrangements. MDAT extends earlier programs which perform pairwise alignments of domain arrangements. MDAT uses a domain similarity matrix to score domain pairs and aligns the domain arrangements using a consistency supported progressive alignment method.

**Conclusion:**

MDAT will be useful for analysing changes in domain arrangements within and between protein families and will thus provide valuable insights into the evolution of proteins and their domains. MDAT is coded in C++, and the source code is freely available for download at http://www.bornberglab.org/pages/mdat.

**Electronic supplementary material:**

The online version of this article (doi:10.1186/s12859-014-0442-7) contains supplementary material, which is available to authorized users.

## Background

Proteins are composed of domains, i.e. amino acid segments which have a specific function and/or a structure, fold independently from the rest of the protein and are evolutionary well conserved [1-3]. Domains are units of evolution, they influence the function of a protein, and can be selected for as a whole [1,4]. The number of known domains is relatively small: currently around 15,000 domains are listed in the Pfam database [5]. About 65-70% of the known proteins contain at least one known domain [6]. However, the number of known arrangements, the combination of domains in a protein, is much higher and steadily and rapidly increasing with more genomes being sequenced [7]. These arrangements evolve over time as domains can be lost, new ones gained and domains are reordered, mostly by gene fusion and terminal domain losses. Typically, rearrangements occur at a rate of tens to hundreds over a span of one million years [8]. Accordingly, rearrangements are more frequent than loss and gain of whole genes, but substantially rarer than changes at the level of amino acids.

Several studies have shown the importance of changes in domain arrangements during evolution. New arrangements can be produced by shuffling of existing domains. These new arrangements played, for example, an important role during the evolution of vertebrates where they are involved in vertebrate specific structures like the cartilage [9]. In addition, it has been proposed that the usage of domains may facilitate convergent evolution. For example it has been shown that netrin and secreted frizzled-related proteins have several independent evolutionary origins [10]. Furthermore, it was proposed that a repository of reusable domains allows for a faster adaptation in plants [8], since a high number of new domains and arrangements in plants are involved in stress and adaption related functions. Changes in domain arrangements are less likely to occur than changes at the amino acid level and are therefore suitable traits for the reconstruction of phylogenies. Accordingly, domain occurrence has been used to calculate large scale phylogenetic trees [11]. Besides these large scale approaches it can be useful to investigate domain arrangements of a single protein family. It has been shown, for example, that the domain arrangements in virulence genes in *Plasmodium falciparum* are probably the result of a trade-off between optimizing within-host fitness and minimizing between-host immune selection pressure [12]. Also, the evolution of Cry toxins is strongly affected by reordering the arrangement of their constituting domains and these rearrangements are important for the virulence of several bacteria [13].

The best currently available methods to study domain arrangements are classical multiple sequence alignment (MSA) methods, for example T-Coffee [14] or Clustal Omega [15]. However, these alignment methods usually do not explicitely take domain arrangements into account and therefore do not incorporate any restriction concerning their alignment. Exceptions are Dialign-Pfam [16] and Cobalt [17] that use domain information to restrict the sequence alignments. Still, none of the existing methods produce a real multiple domain alignment (MDA). An MDA aligns multiple domain arrangements to find the best arrangement of domains using an objective function, similar to the traditional MSA that arranges amino acids and nucleotides.

There are several advantages in using MDAs instead of MSAs. Due to the much shorter arrangement length compared to the primary sequence, an MDA can be calculated faster and with lower memory requirements, which is especially an advantage with large datasets. Another advantage is that a domain arrangement is more conserved than the underlying amino acid sequence. It is therefore possible to produce meaningful MDAs when the amino acid sequences are already too divergent to be compared. Furthermore, it is easier to visually examine the resulting alignments, due to the smaller number of characters.

Since domain arrangement similarity and differences can provide insights into functional similarity and changes between proteins (see above) we present an algorithm which helps to compute an MDA and facilitate the analysis of domain arrangements of different proteins. In this paper, we present MDAT (Multiple Domain Alignment Tool), a program that takes multiple domain arrangements and aligns them. It uses a domain similarity matrix reflecting the similarity between all pairs of domains in the Pfam database. Using a combination of the RADS [18] algorithm and the MSA consistency approach described in T-Coffee [14] an MDA is calculated. The main goal of RADS is to compare and evaluate domain arrangements and to weight differences between domain arrangements. In addition, the resulting MDA can then serve as a backbone structure for the construction of an MSA.

## Results and discussion

### Domain similarity matrix

**Figure 1:**
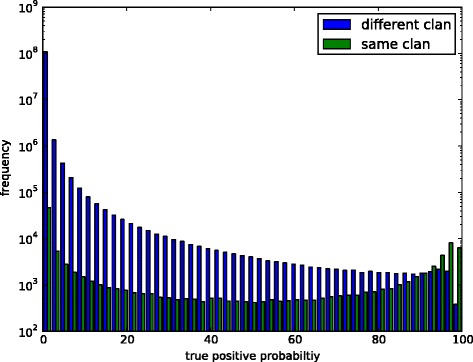
**Domain similarity score distribution.** The scores were calculated by HHsearch, for all pairwise alignment scores of Pfam-A domains (version 27). The values have been divided into two groups depending on whether the two domains belonging to the same clan or not (different or no clan). Values of self alignments are not included.

The Pfam database provides some rough information on domain homology (the “clans”) based on a range of various information evaluated manually [19,20]. Unfortunately, this information cannot be used in an alignment program as clan information is binary only. Therefore, one cannot use clan information reliably to distinguish which domains to match if several possibilities to align a set of domains exits. Another drawback of using clans is that currently only about one third of the almost 15,000 domains in Pfam are associated to a clan. To avoid these drawbacks, we decided to calculate the domain similarity matrix. Figure 1 displays the distribution of match probabilities for each domain pair and how this value relates to being in the same clan or not.

As expected, most domain pairs have a low probability of being a true positive match. It is interesting to note that a high number of domain pairs coming from the same clan have a very low probability of being a true match and that at least some domain pairs from different clans or without clan assignment have a high probability of being a true match.

### MDA

**Figure 2:**
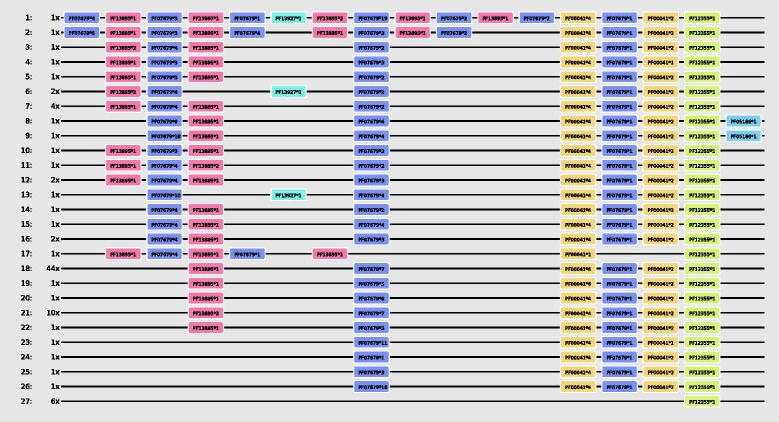
**Example MDA: The IPR021012 family, consisting of 90 sequences is shown.** All segments contain the Dscam domain. The pfam_scan script has been used to perform the domain annotation. The first column depicts the reference arrangement ID, and the second the number of times this arrangement has been encountered.

We use the BAliBASE3 [21] benchmark, which was originally developed for evaluating MSAs, to evaluate the MDAT algorithm. On average 95% of the domain pairs found in the BAliBASE3 benchmark (see Methods) are also found in the test alignments produced by MDAT. Furthermore, the MDAT algorithm is the fastest of all compared methods, even when including the additional step of calculating an MSA from the MDA (see last row in Table 1). An actual example of an MDA is shown in Figure 2. The 90 sequences from the IPR021012 family (Down syndrome cell adhesion molecule, C-Terminal) have been aligned using MDAT after Pfam domain annotation. 27 different domain arrangements based on 6 different domains have been identified. The resulting alignment contains 27 different architectures showing conserved domains, repeat events as well as rare domains in this family.

**Table 1:** **Results of running 3 different methods on the BaliBase3 benchmarks**

	**Set**	**MDA**	**MDA (MSA)**	**Mafft**	**Clustal omega**
	RV11	-	47.78	52.91	59.01
	RV12	-	83.61	89.30	90.60
	RV20	-	80.49	89.31	90.21
SP	RV30	-	67.42	82.50	86.24
	RV40	-	77.14	87.79	90.17
	RV50	-	65.65	84.90	86.20
	Total	-	71.78	81.36	84.00
	RV11	-	24.13	25.76	35.76
	RV12	-	65.02	74.93	78.86
	RV20	-	22.51	31.56	44.95
TC	RV30	-	19.03	42.47	57.50
	RV40	-	33.57	47.98	57.90
	RV50	-	18.56	49.62	53.25
	Total	-	33.09	45.82	55.44
Time (s)	Total	10.37	31.75	92.82	438.08

SP denotes the sum of pairs score, TC the column score. The running times of MDAT includes time for constructing the MDA but not the time for running the domain annotation. The MDAT row shows the time for constructing the MDA only, while MDAT (MSA) shows the time for calculating the MDA with subsequent MSA construction.

### MDA2MSA

**Figure 3:**
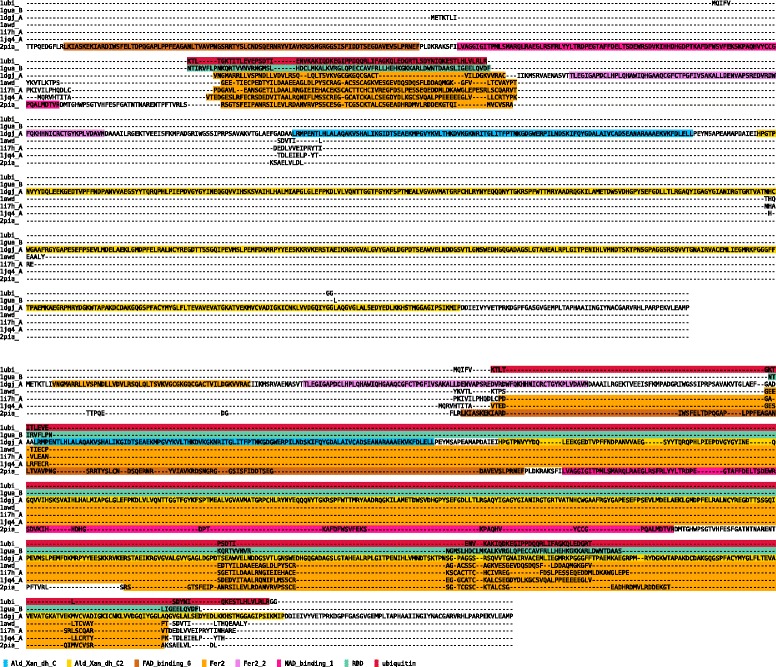
**Example of an MDA based sequence alignment: An MSA of seven sequences from the BAliBASE3 benchmark with annotated domains is shown.** The upper alignment has been generated using MDAT, the bottom one using MAFFT. Due to the incorporation of domain information MDAT is able to align all 5 occurring Fer2 domains correctly together, MAFFT only aligns 4 of the domains and stretches them very widely.

An example for the advantage of using domains in the construction of alignments can be seen in Figure 3. The upper alignment was constructed using MDAT, the lower one using the MAFFT program. Due to the benefits of domain information, MDAT was able to align all five *Fer2* domains together, while MAFFT only aligned 4 of them. Moreover, these domains are not kept as unit but split up into parts that are stretched along the whole alignment. Another example can be viewed in Additional file 1.

The use of domains as anchor-points can strongly reduce the memory usage and running time. Table 1 shows the results of running MAFFT, Clustal Omega and MDAT algorithms on BAliBASE3. MDAT is three times faster than MAFFT and about 9 times faster when calculating only the MDA. The increased speed comes at the cost of accuracy. A combination of different reasons can explain the loss in accuracy. Domain annotations are not perfect and wrongly annotated domains or discrepancies in the boundaries may influence the resulting alignment. Furthermore, an error in the MDA can have a large influence on the resulting MSA as whole regions can no longer be aligned, a problem that all anchor based methods have in common. Additionally, we use a simple implementation of the Gotoh algorithm [22] to perform the sequence alignment; more complex techniques, such as HMMs as used for example in Clustal Omega [15], might provide better results.

## Conclusion

We show that using MDAs themselves has its merits. MDAs can be used to visualize in a simple way the similarity between domain arrangements. Just like any alignment program, MDAT is not able to handle inversions. However, due to the low number of domains in a protein, inversions can be easily detected in a graphical view, which in not possible at the amino acid level. Furthermore, we demonstrate that an MDA is a good starting point for a multiple sequence alignment. It is particularly useful as guidance for the MSA, because it strongly increases the speed with which a multiple sequence alignment is calculated. Currently, the resulting MSAs from MDAT are not as accurate as traditional sequence alignments, however, due to the short calculation time, we are able to handle larger data sets. For many analyses, such as genome projects, the detection of domains is an essential part of the standard annotation procedure. Therefore, domain annotation is often readily available.

## Methods

### Scoring domain matches

Contrary to amino acids, no scoring matrix currently exists to handle domain matches. Therefore, we calculated a domain similarity matrix (DSM) for Pfam-A domains that stores a similarity value for each domain pair. The entries of the DSM are calculated using the HHsearch [23] program. Every HMM model of a domain in Pfam is aligned with every other HMM model in Pfam resulting in 14831^2^ alignment pairs. As recommended [23], we used the probability of a true positive match as a similarity score and not the e-value. A true positive match value corresponds to the probability that the two models compared belong to homologous sequences or if the sequence alignment supports a good structural alignment. Contrary to the standard BLOSUM [24] and PAM [25] matrices, the DSM contains only positive values between 0 and 100. The huge majority of entries in the DSM are values below 1, corresponding to domain pairs without similarity. Accordingly, these values do not need to be stored and can therefore be removed from the matrix without loss of information, thus reducing the actual size of the DSM.

### MDA construction

The MDA is constructed in several steps and is based on the RADS [18] pairwise and the T-Coffee [14] consistency aligner.

1. Domain collapsing: Given a set of domain arrangements, the first step of the MDA construction is to collapse identical domain arrangements into a single one. A set of identical domain arrangements is from here on represented with a single arrangement. The length of a domain in this representative arrangement is defined as the average of the domains it contains. A change in tandem domain repeats is the most frequently occurring domain rearrangement event [4]. Tandem repeats are very similar and their correct alignment on the domain level difficult to achieve. We therefore collapse successive repeats of the same domain into a single one as previously proposed [26]. This facilitates the alignment process that can be easily confused by a high number of near identical domain matches introduced by repeat copies.
2. Library construction: In the next step the RADS algorithm, a dynamic programming algorithm, is used to produce alignments between all pairs of arrangements. RADS has been extended to use the DSM to score a match of two domains instead of a fixed value. The matches identified in this alignment are then stored in a library.
3. Library extension: The matches from the library are rescored according to the algorithm described in T-Coffee. The reweighting has the purpose to increase the score of a match that is supported by matches in a third sequence: If domain *α* in arrangement X and domain *β* in arrangement Y are matching, then the score of the match *α*−*β* is increased if there are arrangements Z with domain *γ* that is matching *α* as well as *β*.
4. Alignment calculation: Using these scores a normal progressive alignment, as first described by Higgins and Sharp [27], is performed.
5. Refinement: The last step of the algorithm is a simple refinement step. Blocks of domains are shifted to other columns if this increases the number of identical domains in a column.

### MDA2MSA

**Figure 4:**
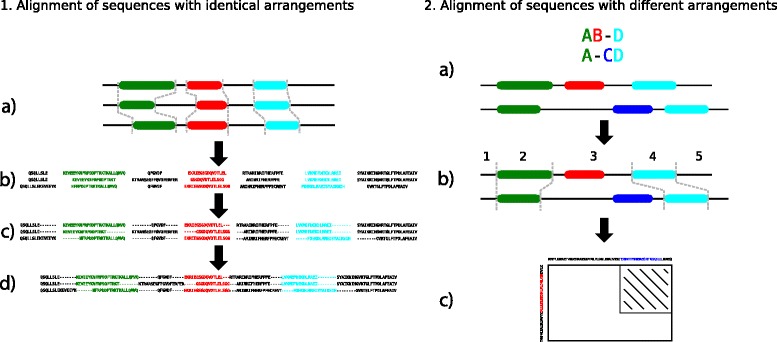
**Workflow of the MDA2MSA algorithm.** Step 1: Sequences with identical domain arrangements **(a)** are split according to domain boundaries **(b)**. Then each part is separately aligned **(c)** and finally all parts are merged back together **(d)** into a single alignment. Step 2: The MDA **(a)** is used as a guide. The sequences are split into parts according to the MDA **(b)**. Cuts are performed at the borders of aligned domains resulting in 5 parts. Each pair of sequence segments can now be aligned separately. In case unaligned domains occur in the MDA (part 3), the dynamic programming algorithm is changed such that it maintains the order of domains **(c)**. The striped area represents the area that is not calculated because the MDA forbids the alignment of the two domains.

An MDA can be used to guide the MSA alignment process. MDAT uses a similar approach to the one described in Dialign-Pfam. Blocks of domains are used as anchor points to limit the search space and thereby increasing the speed of the alignment calculation. Furthermore, using the MDA as an anchor, guarantees that the correct domains are aligned with each other (see Results and discussion). Unlike Dialign-Pfam, MDAT uses a completely automatic approach and because we use the MDA as guide, the anchors do not need to be of a single domain type. The MDA is used as a backbone for the MSA construction. This process can be divided into two major steps. In the first step, sequences with identical domain arrangements are aligned, in the second the alignments of the first step are aligned with each other. This two-step process is shown in Figure 4.

First alignment step: All sequences that are represented by the same domain arrangement are aligned first. The sequences are split at domain boundaries and each pair of segments is aligned separately, thus allowing easy parallelization of this step. Domain segments are aligned using a banded alignment approach as previously described in the Pecan genome aligner [28].

Second alignment step: Following a guide tree constructed from the domain architecture similarity, the alignment profiles computed in the first step are progressively aligned with each other. At each node in the tree, two profiles are aligned that are based on different domain architectures. Similar to the first step, sequences can be split at domain boundaries. However, this is only possible at domains that are aligned with each other. The sequence segments between two aligned domains cannot be simply aligned in a global fashion, because there may be non-aligned domains which should not be aligned on the sequence level either (see Figure 4). In this case, the corresponding area in the dynamic programming matrix is declared forbidden. The alignment algorithm does not pass through these areas that are forbidden by the MDA and thus avoids violating the order of the domains as defined by the columns of the MDA.

### Benchmarking

Currently no reference benchmark exists for the evaluation of MDAs. Therefore, we use the BAliBASE3 [21] benchmark that was originally developed for MSAs. To be able to evaluate an MDA, we annotated the sequences included in BAliBASE3 with Pfam domains using pfam_scan [5] in combination with the HMMER3 [29] program. Since BAliBASE3 is a set of reference sequence alignments it is possible that more than one domain is aligned to another one, conflicting with the alignment definition that a domain is aligned only to a single other domain. We define two domain as being aligned to each other if at least three quarters of both domains are aligned with each other. For this benchmark the repeat-collapsing has been turned off to allow a comparison of the MDA with the pairs extracted from BAliBASE3.

To check the performance of the MDA2MSA algorithm, MDAT has been run on the BAliBASE3 benchmark and has been compared to two other alignment methods MAFFT (v6.940b) [30] and Clustal Omega (v1.2) [15].

## Additional file

Additional file 1
**Example of MDAT sequence alignment.** PDF showing another example where the usage of domain information improves the alignment.
