# Supplementary material for: MDAT- Aligning multiple domain arrangements
Source: BMC Bioinformatics. 2015 Jan 28;16(1):19. doi: 10.1186/s12859-014-0442-7 (PMC4384290; doi:10.1186/s12859-014-0442-7)

Alignment of the BB40023 dataset. The alignment on the left has been computed using MDAT, on the right using MAFFT.

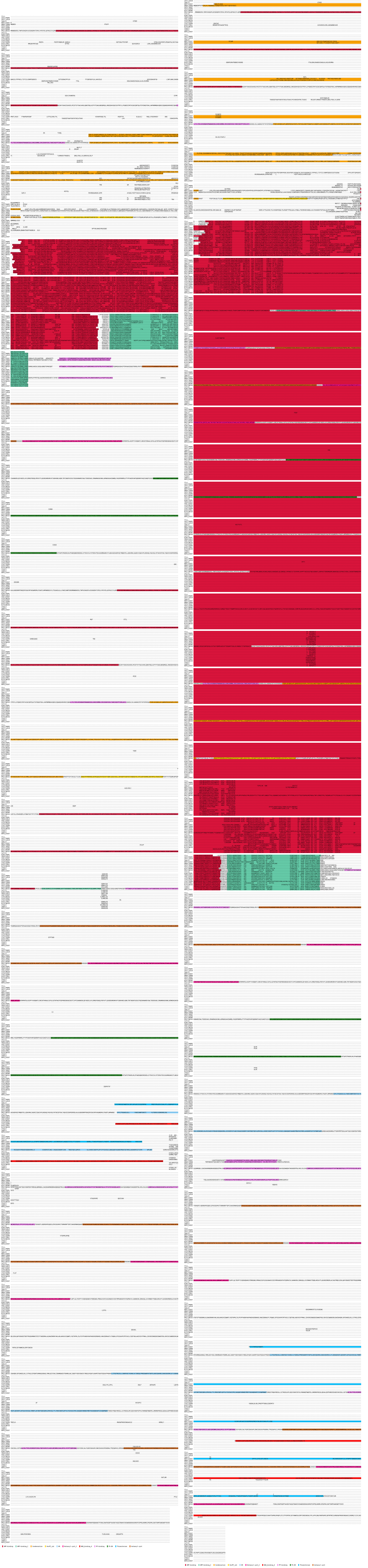

Supplement: Additional file 1 — Example of MDAT sequence alignment. PDF showing another example where the usage of domain information improves the alignment. [file 12859_2014_442_MOESM1_ESM.pdf]
